# Statecraft at the frontier of capitalism: A grounded view from China

**DOI:** 10.1177/03091325241268953

**Published:** 2024-08-12

**Authors:** Fulong Wu, Handuo Deng, Yi Feng, Weikai Wang, Ying Wang, Fangzhu Zhang

**Affiliations:** 4919 University College London, UK

**Keywords:** capitalistic logic, China, governance, statecraft, state entrepreneurialism, territorial logic

## Abstract

The death of urban entrepreneurialism is proclaimed surprisingly by opposite conceptualisations of austerity urbanism and radical municipalism. This paper argues that rather than seeing them as contrasting types, post-pandemic statecraft reflects the increasing tension and entanglement between capitalistic and territorial logic. From the ground of Chinese urban governance, we illustrate how Chinese statecraft maintains state strategic and extra-economic intention through deploying and mobilising market and society – to create its own agents and to co-opt those that are already existent or emerging. This statecraft is illustrated through community building, urban development, and regional formation.

## I Introduction

The salient role of the Chinese state in urban governance seems to be regarded as exceptional. However, arguably, with a shift to new state capitalism, urban entrepreneurialism has been proclaimed to come to an end by austerity urbanism (Davidson and Ward, 2014; Peck, 2012, 2017), municipal or city statecraft (Lauermann, 2018; Pike et al., 2019), and radical municipalism (Roth et al., 2023; Thompson, 2021). Surprisingly, these perspectives have opposite theoretical positions in terms of the dynamics of capital accumulation. Austerity urbanism foresees greater capital influences and capture over urban governance (Peck and Whiteside, 2016), while new municipalism suggests an alternative picture of the transformative role of social and cooperative movement and proximity in resisting financialised governance and rentierism (Penny, 2022; Thompson, 2021). They present different typologies, resonating with a long tradition of the varieties of capitalism (VoC) (Hall and Soskice, 2001). Beyond VoC, Peck and Zhang (2013) argue that capitalism and its state form could be combined in a variegated way. They raise a profound question about ‘the extent to which the Chinese economy can be meaningfully characterised as capitalist’ (357). Their question has a theoretical implication beyond China as it reveals the limit of ‘conventional understandings of capitalist variety’ (357). With increasingly state-interventionist roles being identified worldwide, we examine contemporary Chinese statecraft to demonstrate this general trend with neighbourhood, city, and regional statecraft studies and reveal its nature in globally universal dynamics of capital accumulation.

Following Peck’s (2021) notion of capitalism’s cusp, we develop a grounded view from China to interrogate its statecraft at the frontier of capitalism. The notion of the frontier has two implications. First, China is at the forefront of experiencing geopolitical tensions in global capitalism. Understanding its governance changes is crucial for comprehending urban governance in contemporary capitalism. Second, the role of the state has always been prominent in China’s urban governance. Although the tension between capitalistic and territorial logics has long been universal in governance, their clash in the recent decade has given rise to various forms of new governance experiments globally, particularly under the banner of statecraft. In this vein, research on China’s statecraft is not only about China’s specific form of governance but also about how far a state can go to sustain capital accumulation and achieve sovereignty. This indicates capitalism’s territorial limit even in its state form (Arrighi, 2007; Polanyi, 1944). The frontier means both geographical reach and distance to a mature state. It indicates a pervasive capitalistic logic, but capitalism is yet to come (Wu, 2022). By this we mean China is a frontier of global capitalism.

Being a non-capitalist economy, China has a path dependence on prioritising territorial sovereignty in statecraft, even with a long tradition of market economy (Arrighi, 2007). The national-state has no fundamental intention for capitalist profit motivation (Weber, 2023). The capitalist class and interest groups have been secondary in politics. Recent scholarship understands it as ‘market in state’ (Zheng and Huang, 2018) to see how different social forces are filtered through the state. On the other hand, capitalistic logic has transferred and penetrated across borders and scales since the world-scale expansion of capitalism (Alami and Dixon, 2023; Arrighi, 1994; Harvey, 2003; Weber, 2023). This makes the dynamics of capital accumulation crucial in studying Chinese statecraft.

Statecraft has become increasingly popular in conceptualising urban governance because the state has taken more visibly proactive roles in economic and extra-economic management. The concept was initially defined by Bulpitt (1986: 21) as ‘the art of winning elections and achieving a necessary degree of governing competence in office’ in the 1970s UK. It referred to the ways state actors, whose goal was to stay in office, managed affairs. The political dimension was emphasised. China’s statecraft resembles this political dimension because the continuing power of the Chinese Communist Party (CCP) is an overwhelming consideration. Neither the political definition of ‘statecraft’ (Bulpitt, 1986) nor the economic structural explanation of urban entrepreneurialism (Harvey, 1989) works perfectly in unravelling diversified statecraft. Pike et al. (2019)’s city statecraft and Lauermann (2018)’s municipal statecraft already reflect a mix of capitalistic and territorial logics.

Recently, statecraft has been increasingly used as a conception of governance (Kutz, 2017; Lauermann, 2018; Pike et al., 2019). Pike et al. (2019: 4) use statecraft to analyse ‘forms of governance at the city, city-region, metropolitan and local scales as well as in non- or para-state spaces’. In studying financing urban infrastructure development, city statecraft is understood as ‘the art of government and management of state affairs and relations (79)’, which ‘seeks to understand and explain how local government councillors and officers are engaging with finance actors and the financialization process’ (Pike, 2023: 14). Lauermann (2018: 206) identifies ‘a more interventionist role for municipal state institutions’ in municipal statecraft that ‘draw[s] on entrepreneurial toolkits to pursue a more diverse portfolio of investments and agendas, in parallel to pursuing growth’. He delinks entrepreneurialism with neoliberal growth politics. The statecraft does not necessarily take the entrepreneurial form of governance. This differs from Harvey’s (1989, 2007) conceptualisation of neoliberalism as a strategy for class restoration and maintaining accumulation. Although the state plays a crucial part in neoliberalism, the capitalistic logic is the underpinning driving force (Harvey, 2005). In contrast, multiple perspectives argue that entrepreneurial statecraft is still frequently used today in speculative and experimental ways (Lauermann, 2018; Phelps and Miao, 2020; Wu et al., 2022).

This suggests that entrepreneurialism is not always tied to neoliberalism or capitalism. Instead, it applies to various regimes and forms of governance with different policy agendas. While Schumpeter’s definition of entrepreneurism refers to securing surplus profits through innovation in contemporary capitalism, Jessop and Sum (2000) extend the concept of entrepreneurialism to the state as innovative strategies to maintain or enhance its economic competitiveness compared to other cities and economic spaces. These strategies are ‘formulated and pursued in an active, entrepreneurial fashion’ (2289). They extend the toolkits of Harvey’s ‘urban entrepreneurialism’ as a particular mode of neoliberalism. Following this direction, thinking entrepreneurialism beyond assumed (pro-)market behaviour, this paper highlights innovation in statecraft and experimental governance to advance extra-market objectives and intentions (Sun et al., 2023). Entrepreneurialism can refer to the speculative action for maximising profits and expanding accumulation (Beswick and Penny, 2018; Ferm and Raco, 2020; Harvey, 1989; Peck, 2012). It can also refer to more innovative and experimental approaches (Jessop and Sum, 2000) that deconstruct existing models and achieve multiple objectives (Lauermann, 2018; Mazzucato, 2013; Sun et al., 2023; Wu, 2018). Entrepreneurs can be states, organisations and individuals operating in market or non-market contexts (He et al., 2018; Jessop and Sum, 2000; MaFarlane, 2012; Mazzucato, 2013). When applied to individuals or social groups, entrepreneurial subjectivity refers to a motivated autonomy of self-responsibility and self-organisation in everyday life (Jessop, 2022; MaFarlane, 2012; Zhang and Ong, 2008). As the state plays a critical role in entrepreneurial activities, which is proclaimed to strengthen (Lauermann, 2018; Pike et al., 2019) or transform (Roth et al., 2023; Thompson, 2021), it is imperative to re-examine versatile entrepreneurial statecraft systematically in the changing political economy.

**Figure 1.**
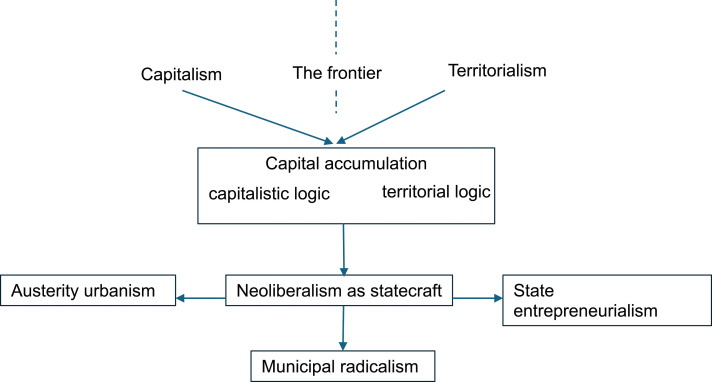
A variety of statecraft in capital accumulation.

To understand ‘the evolutionary nature of the modern world system’, Arrighi (1994: 33) argues that ‘central to such an understanding is the definition of “capitalism” and “territorialism” as opposite modes of rule or logics of power’. Inspired by Arrighi, Harvey (2003: 183) points out a specific form of capitalism – the new imperialism which ‘arises out of a dialectical relation between territorial and capitalistic logics of power’. He stressed that ‘the two logics are distinctive and in no way reducible to each other, but they are tightly interwoven’ (183). He explicitly defines the two logics, as ‘by territorial logic, I mean the political, diplomatic and military strategies invoked and used by a territorially defined entity such as a state’, and ‘the capitalistic logic focuses on the ways in which economic power flows across and through continuous space’ (Harvey, 2006: 107). By separating these two logics, he points out that ‘a central contradiction exists’, which ‘is internalized within capital accumulation’ (107). However, Jessop (2006: 157) criticises that ‘the asymmetrical conceptual development of the two logics leads him to privilege the capitalist logic of power in both his theoretical and his empirical analyses’. In this paper, state entrepreneurialism is raised to stress the contradiction of the two logics but further rebalance their treatments at the frontier of ‘capitalism’ and ‘territorialism’ (Figure 1). Neoliberalism is a form of statecraft. Austerity urbanism and state entrepreneurialism represent two opposite directions of post-2008 responses to the global financial crisis. The former continues to enlarge the private sector and the latter relies increasingly on state-interventionalist roles (Figure 1). Contextualising two logics in urban studies, we explore capital accumulation and territorial politics in statecraft (Table 1).

**Table 1.** Capitalistic and territorial logics in statecraft: General definition and specific presentations in China’s residential, city and regional statecraft.

	Capitalistic logic	Territorial logic	Contradictory relationship
In general	The endless desire to maximise profits and expand accumulation spaces	A political group maintaining sovereignty within a territoriality, or a political leader aspiring to stay in office or get promoted	One cannot survive without the other (Arrighi, 1994) Their natures fundamentally contradict each other (Harvey, 2003)
	Universal and prevailing worldwide	Addressing issues and protecting collective interests bound by territorial boundaries, including neighbourhoods, municipalities, and national-states	
		Shaped in historical and geographical materialities	
In China	Share the general capitalist logic	Path-dependent in Chinese history, with particularity	
	After the Opium War in 1840: An intrusion of capitalist market logic into the Chinese economy	Modernisation as a national-state strategy, CCP centrality, and upward accountability politics	
	After the 1970s: China proactively embraced economic globalisation		
City	Land-backed local borrowing and financial techniques	CCP cadre promotion system: Upward accountability	Alignment: City government pro-growth strategies
	Maximizing revenue	Central state initiatives: ‘Ecological Civilisation’, ‘People’s city’, and ‘common prosperity’	Contradiction: Preserving the ecological quality regardless economic costs, local government debt
	Economic growth		
Neighbourhood	The privatization of housing and the marketisation of neighbourhood public goods provision (as delivered by property management company and coordinated by homeowner’s association)	Local cadres may get promoted through ‘innovation tournament’ and governance experiments Addressing disputes, contestations, protests, and social conflicts – issues threatening social stability and state centrality The grassroots party leadership	Alignment: Local cadres encourage ‘enterprising self’ and mobilise NGOs to cultivate governable individuals and communities
	‘Enterprising self’: Mobilising individuals, neighbourhood groups, and social organisations to become de facto entrepreneurs in everyday life		Contradiction: Marketisation and privatization fail to build social trust Residents refuse to act like ‘enterprising self’ and govern themselves. The local state has to turn to party-led community building
Region	Agglomeration for economic growth Spatial restructuring as a spatial fix addressing the accumulation crisis	Rezoning to consolidating state management Coordinated development in cross-boundary areas, policy experimentation, and solving social and ecological issues Tackling changing geopolitics	Alignment: Geopolitical considerations and economic restructuring desires co-shape new regional plans
			Contradiction: The tensions between the envisioned territorial coherence and uneven development, between the state-control/led development and capital mobility, and between the pursue of non-economic territorial agendas and territory-based economic growth

As is unpacked in section 2, the endless desire to maximise profits and expand accumulation spaces has driven the urban process of capital accumulation (Harvey, 1989). On the other hand, territorial politics involves political groups or leaders wanting to stay in office (Bulpitt, 1986; Guo, 2020; Pike, 2023) and the logic of guaranteeing collective interests bound by territories (Arrighi, 1994; Jessop, 2006; Thompson, 2021). Capitalistic logic is footloose, while territorial logic is spatially fixed. However, capital accumulation is maintained through statecraft. It resonates with the complex nature of the capitalist state (Peck and Zhang, 2013) – including both territoriality (Brenner, 2019; Jonas and Moisio, 2018) and capitalist dimensions (Harvey, 2003, 2007). It also works beyond the capitalist context because of a pervasive global capitalism system nowadays. As Su and Lim (2023) suggest, a sovereignty–accumulation nexus has underpinned Chinese statecraft since 1949.

The entanglement and tension of capitalistic and territorial logics in capital accumulation are universal, while their specific combination into statecraft is particular to a locality out of the historical process. The intertwining nature of contingencies means a possible wide spectrum of statecraft, ranging from neoliberalism (Harvey, 1989), the entrepreneurial city or state (Jessop and Sum, 2000; Mazzucato, 2013; Phelps and Miao, 2020), austerity urbanism (Peck 2012), an ‘elite capture’ in late capitalism (Lauermann and Mallak, 2023), to state entrepreneurialism (Anguelov et al., 2023; Cartier, 2018; Pearson et al., 2023; Wu, 2018) (Figure 1).

The intensified contradiction, as revealed in geopolitical conflicts and system-wide crises, exacerbates the transformation of statecraft as a global trend. Since the 1970s, state intervention has adopted different forms (e.g. de-regulation, re-regulation) supporting capital accumulation (Brenner, 2019; Jessop, 2006). Recently, state-interventionist roles have been increasing (Su and Lim, 2023; Wu et al., 2022; Wu and Zhang, 2022). Particularly in post-pandemic times, escalating geopolitical tensions trouble global circulation and steer statecraft more assertively (Yeung, 2023).

Our grounded view of Chinese statecraft is clarified in the contradiction between capitalistic and territorial logics at this historical conjuncture. Through examining community building, urban development, and regional formation, we reveal that Chinese statecraft maintains state strategic and extra-economic intention by deploying and mobilising market and society. The statecraft incorporates versatile pragmatic approaches and combinational or intertwined modalities (Ekers et al., 2012; Keil, 2018; McNally, 2020; Peck, 2021; Robinson et al., 2022). It illustrates the universal tension between the two, thus conceptually connecting various statecrafts. We conceptualise this grounded view as evolving state entrepreneurialism, a timely refining argument of ‘planning centrality, market instruments’ (Wu, 2018). In post-pandemic times, state entrepreneurialism sees a stronger state’s role (Wu, 2020, 2022; Wu and Zhang, 2022; Zhang and Wu, 2024). It aligns with the global trend of statecraft but is shaped by Chinese historical materiality.

This paper is organised as follows. The next section reviews a variety of statecraft worldwide. Section 3 explains the aim of conceptualising Chinese statecraft as state entrepreneurialism. It explains how the grounded view incorporates both capitalistic and territorial logics. Then, sections 4–6 present versatile governance approaches in Chinese statecraft in the cases of cities, neighbourhoods, and regions. Section 4 explores how municipal governments develop, deploy, and remould market-like operations to internalise market to achieve extra-economic goals. In section 5, we move downwards to residential statecraft and explore how the Chinese state mobilises and co-opts individuals and social agencies to consolidate party-state governance through experiments in community participation. Similarly, in section 6, we move toward regional statecraft and explore how states employ re-scaled regional planning and concrete zonal and cross-boundary projects to orchestrate intra-state relationships and conflicting interests for geopolitical and state-intentional goals. Finally, we conclude with the nature and implications of Chinese statecraft.

## II Statecraft: A dialectic of capitalistic and territorial logics

We explore concepts of statecraft, considering the capitalistic and territorial logics. In brief, urban entrepreneurialism (Harvey, 1989), austerity urbanism (Peck, 2012), and post-political governance (Swyngedouw, 2009) reflect more of a dominant capitalistic logic. In contrast, authoritarian (Kinossian and Morgan, 2023; Zhang, 2012), party-statecraft (Anguelov et al., 2023), new municipalism (Roth et al., 2023; Thompson, 2021), and politically-oriented statecraft (Bulpitt, 1986; Guo, 2020) has a dominant territorial logic. The two logics are combined into more complex statecraft, such as new state capitalism (Alami and Dixon, 2023; Sperber, 2022; Su and Lim, 2023), geo-economic or municipal statecraft (Kutz, 2017; Lauermann, 2018; Phelps and Miao, 2020). As will be shown, even the most ‘extreme’ forms of statecraft of either capitalistic or territorial logic must rely on the other to sustain. Their interdependence explains the intertwined nature of statecraft. However, often neglected is their inherent tension. This is reflected by rising territorial logic influencing state-interventionist roles in statecraft. Interventionist state strategies are rediscovered in various geographies (Cirolia and Harber, 2022; Penny, 2022; Roth et al., 2023; Russel, 2019).

The capitalistic logic of maximising profits and expanding accumulation spaces is a significant dimension of statecraft. From a totality view of capitalism, ‘the deep-seated capitalist transformations’ have ‘underpinned the historic arc in the trajectories of state intervention on a global scale’ (Alami and Dixon, 2023: 87). Driven by capitalistic logic, the actors can be state and non-state organisations, enterprises, and individuals (Arrighi, 1994; Bremmer, 2010; Harvey, 2003; Sperber, 2022; Zheng and Huang, 2018). Capitalist players are footloose and not confined by territoriality (Harvey, 2003). In such a context, Harvey’s (1989) urban entrepreneurialism describes the ‘urban process of capital accumulation’ in contemporary capitalism. However, the concept also involves territorial logic, because the urban state has become a major actor in attracting capital and businesses (Harvey, 1989). The local states promote public-private partnerships and attract footloose industries in the fierce global competition for capital. As a statecraft, urban entrepreneurialism is a pro-accumulation strategy for growth. Similarly, post-political technocratic governance (Fearn and Davoudi, 2022; Swyngedouw, 2009) illustrates how such strategies marginalise social welfare and inclusion goals by focusing on financial sheets and technical details. After the 2008 global financial crisis, austerity urbanism (Peck, 2012: 626) enforces this neoliberalism in post-crisis times as ‘a necessary response to market conditions’. They are the statecraft more dominated by capitalistic logic (Ferm and Raco, 2020; Kirkpatrick and Smith, 2011; Weber, 2010), mostly in the neoliberal capitalist context.

Territorial logic demonstrates territorial-bounded interests associated with individuals (often political leaders), neighbourhoods, municipalities and national-states. First, a political group maintains sovereignty within a territoriality, or a political leader aspires to stay in office or get promoted. It is universal across political contexts. Bulpitt (1986) studied statecraft in the UK’s democratic electoral politics. The Conservatives promoted its neoliberal reform to win votes and get elected. Miao et al. (2023) suggest that policy priorities must be carefully decided to maintain political leadership in national and local states. They cannot be reduced to a single economic objective. In China’s upward accountability politics, Guo (2020) argues that a local state leader’s ambition of getting promoted is achieved through developing the local economy and meeting other assessment criteria. In authoritarian states (Anguelov et al., 2023; Kinossian and Morgan, 2023; Zhang, 2012), the party or state’s central intention is to maintain sovereignty. The concrete tools adopted evolve with changing political-economic contexts (Su and Lim, 2023).

Second, territorial logic involves addressing issues and protecting collective interests bound by territorial boundaries, whether they are national-states, municipalities, or neighbourhoods. Arrighi (1994) argues that the state has harnessed capital for territorial expansion in historical capitalism. In the periphery of capitalism, often the former colonial or semi-colonial territories of imperial capitalism, territorial logic is often reflected in a national-state’s collective intention to modernise and catch up with the West (Weber, 2023; Wu, 2022, 2023). In municipalities, territorial logic involves guarding collective interests and well-being through different organisational bodies or movements (Jessop and Sum, 2000). When collective interests deviate from pro-accumulation and become primary in politics (Janoschka and Mota, 2021; Thompson et al., 2020), it indicates a dominating territorial logic in statecraft. For example, an emerging body of new municipalism literature approaches the municipality as a ‘strategic entry point’ for ‘radically democratic politics’ (Russell, 2019: 996; Angel, 2021), contrasting sharply with the neoliberal growth agenda. The agenda of new municipalism involves pro-democracy, local autonomy, anti-austerity, anti-eviction, equalities, and spatial justice (Janoschka and Mota, 2021; Thompson et al., 2020) by applying innovative and, sometimes, radical approaches (Roth et al., 2023). Social movements and local autonomy mobilise ‘not on post-political policy mobilities but on urban solidarities in contesting neoliberal austerity urbanism and platform capitalism’ (Thompson 2021: 317). In governing residential neighbourhoods, ‘socially-engaged’ municipal statecraft represents a tool the municipality uses to bridge social and economic agendas through social collaboration (Teo, 2023; Wang et al., 2024b).

Most statecrafts incorporate both territorial and capitalistic logics in a more balanced combination. They harness each other for their ends. Inspired by strategic selectivity (Jessop, 2007, 2016), a particular state as an institutional ensemble is more open to a set of statecraft than others. The selectivity, reflecting a particular intertwining of the two logics, is contingent on historical and geographical conditions (Peck, 2017, 2023).

Frist, the territorial logic often aligns with or centres on capital accumulation. This can be an entrepreneurial city as a territorially bound growth machine (Harvey, 1989). The ‘coercive law’ (Harvey, 1989) of capitalistic logic drives urban states to attract industries and investments in zero-sum competition (Weber, 2010). It may also motivate entrepreneurialism within public institutions to enhance territorial strength in global competition (Mazzucato, 2013; Miao and Phelps, 2022). The pervasive capitalistic logic penetrates different political contexts through economic globalisation. Instead of an ideological contrast between ‘liberal Hong Kong’ and ‘authoritarian Singapore’ (Zhang, 2012), authoritarian neoliberalism indicates dominant state control can be utilised to promote capital accumulation and achieve capitalist class restoration – through accumulation by dispossession (Harvey, 2005). Similarly, new state capitalism literature depicts how national-states use territorial power to maintain accumulation and outcompete their peers in global capitalism (Peck, 2023). Lim (2017) argues that post-reform China shifts territoriality from national-state to city-regional for the sake of capital accumulation. Among national-states, Rolf and Schindler (2023: 2) show ‘Beijing and Washington instrumentalise and mobilise domestic platform firms in pursuit of geopolitical–economic objectives’. At the municipal level, geo-economic statecraft arises as a ‘deliberate strategy of economically oriented geopolitics designed to manage the contradictions of global capital mobility’ (Kutz, 2017: 1226). In response to the 2008 global financial crisis and, more recently, the COVID-19 pandemic ramifications, pragmatic municipal approaches (Aldag et al., 2019; Warner, 2023) and state-led financialised governance (Christophers, 2019) have become more common in crisis management, in contrast to austerity urbanism response wherein local states cut services (Peck, 2012).

Second, territorial logic of power harnesses capital accumulation in turn. Unlike the neoliberal growth machine, Jessop and Sum (2000) view Hong Kong’s entrepreneurialism as a city-state strategy. Moreover, party-state capitalism is unlike new state capitalism, because the party-state uses capital to maintain party sovereignty (Pearson et al., 2023). The central intention is no longer capital accumulation but reflecting a dominating territorial logic in decision making, sometimes at the sacrifice of capitalist interest groups. Recent debates regarding new state capitalism (Alami et al., 2022; Peck, 2023; Sperber, 2022) show how capitalistic and territorial logic reinforces with each other. A similar intertwining relation is found in municipal statecraft, where the state-interventionist roles use entrepreneurial toolkits to achieve diversified municipal-based goals, mixing economic and extra-economic purposes (Lauermann, 2018).

Although interdependent, capitalistic and territorial logics represent two fundamentally different aspects inherently in tension. Territoriality is not always related to a pro-accumulation growth agenda (Wu et al., 2022). Neither does it necessarily lead to local autonomy or social equity (Russell, 2019). There is a fundamental contradiction between an ‘endless’ expansion of capital accumulation space and a ‘comparatively stable organization of political space’ (Su and Lim, 2023: 701). The contradiction has intensified today.

Statecraft has been increasingly shaped by territorial logic in a way that contradicts capitalistic logic. Global competition among national-states and city-regions over technological industries increasingly restricts capital investment mobility (Rolf and Schindler, 2023; Yeung, 2023). Radical municipalism foresees a bleak future for state politics under capitalism and calls for bottom-up urban movements in municipal-based collective actions (Roth et al., 2023; Thompson, 2021). In China, over-accumulation in property-led (re)development and land financialisation threatens political and social stability (Feng et al., 2022; Wang et al., 2022b). The state’s restricting debt rate for maintaining governance capacity constrains the capitalist logic of financial borrowing (Li et al., 2023b; Pan et al., 2017). Even though the local state tends to align with capitalistic logic, the central state follows a territorial logic that controls capital’s ‘negative role’ (Pearson et al., 2023). Similarly, the state has deemed it imperative to protect the ecological environment and arable land for maintaining governance and has tightened development restrictions (Kostka and Zhang, 2018; Zhang, 2021). This hinders land-driven capital accumulation. There is a global trend of statecraft as the contradiction between territorial and capitalistic logics intensifies.

## III Chinese statecraft of state entrepreneurialism

The initial notion of state entrepreneurialism is a tactic experiment of Harvey’s (1989) conceptualisation of urban entrepreneurialism. However, the grounded view does not support the centrality of capitalist class restoration in China as in Harvey’s (2007) neoliberalism because China is at the frontier of capitalism. The ‘frontier’ indicates a pervasive capitalistic logic, but the state is not capitalist due to the state’s intention to deviate from capitalistic logic. The notion of the frontier is not exceptional to China (Arrighi, 2007; Polanyi, 1944) and may apply to other peripheral areas of global capitalism. China shows path-dependent territorial politics, causing a particular intertwining of capitalistic and territorial logic. There has been an intrusion of capitalism into the Chinese economy since the Opium War in 1840. In the 1970s, approaching the end of the Cold War, China proactively embraced economic globalisation (Wu, 2022). A coherent territorial logic from the late Qing reformers to the CCP is to create a modern nation and catch up with the West (Weber, 2023). Capitalism was used for national modernisation. It has combined closely with the CCP’s territorial logic of maintaining centrality. This centrality is enabled at the local level through a party-state bureaucratic system. The cadre evaluation system and upward accountability urge local leadership to align with central political mandates (Guo, 2020; Li and Zhou, 2005), as shown by local leadership’s desire to get promoted and manage crises through interterritorial competition and innovative experiments (Lin et al., 2022; Teets et al., 2017).

Modernisation as a national-state strategy, CCP centrality, and upward accountability politics portray territorial logic in Chinese statecraft. It shares commonalities with other contexts in terms of maintaining party or individual leadership (Ayres et al., 2018; Bulpitt, 1986) and national interests (Rolf and Schindler, 2023). However, it differs from active collective actions based on municipalities and neighbourhoods (Vincent, 2023) in new municipalism (Thompson, 2021). At this frontier of capitalism, our conceptualising Chinese statecraft is state entrepreneurialism: Chinese statecraft maintains state strategic and extra-economic intention through deploying and mobilising market and society – to create its own agents and to co-opt those that are already existent or emerging. From examining statecraft in neighbourhoods, cities, and regions, this is a more up-to-date and refined argument than ‘planning centrality, market instruments’ (Wu, 2018).

First, the refined view shows a particular historical materiality. It explains how the state’s strategic intentions are conditioned and constrained by capital accumulation and how it utilises and mobilises the conditions for achieving strategic objectives. This combination highlights the state’s intentionality while utilising and being constrained by capital accumulation (Deng, 2023). State entrepreneurialism understands entrepreneurialism as statecraft – provincialising ‘urban entrepreneurialism’ as its possible particular statecraft in its political context – denoting a particular form of combining two logics with tensions therein. Because of the frontier of capitalism, capital accumulation is not the ultimate goal but rather a means of achieving state strategic intentions and enhancing governance capacity. Therefore, state entrepreneurialism differs from mainstream new state capitalism, which intends to focus on capital accumulation using territoriality as an instrument (Alami et al., 2022; Peck, 2023; Sperber, 2022). On the other hand, state entrepreneurialism does not perceive the party-state could transcend the existing economic structure. This differs from authoritarianism and party-statecraft perspectives focusing on the dominating (party-)state control over capital (Kinossian and Morgan, 2023; Kostka and Zhang, 2018; Pearson et al., 2023). In contrast, state entrepreneurialism emphasises economic constraints and tendencies towards crisis. Realising state strategic goals requires mobilising various market and social actors.

Second, the refined view demonstrates an even stronger state’s role in post-pandemic times, increasingly influenced by geopolitical tension and domestic issues threatening state capacity (Rolf and Schindler, 2023; Su and Lim, 2023; Wu et al., 2022). Political mandates are increasingly overcoming maximising profits and expanding accumulation in urban projects, including Ecological Civilisation, high-quality development, and ‘People’s City’ (Kostka and Zhang, 2018; Li, 2022; Li and Zhong, 2021; Zhang and Wu, 2023). Local governments deliver environmental projects, social infrastructures, and innovation parks (Lin et al., 2022; Pow, 2018; Shen et al., 2020; Wang et al., 2022; Zhang and Lan, 2023; Zhang and Wu, 2024; Zhu et al., 2024), which resembles city or municipal statecraft and innovative governance approaches in late capitalism (Lauermann, 2018; Phelps and Miao, 2020; Pike et al., 2019). However, the role of the state differentiates Chinese statecraft from these mutations of municipal statecraft. Unlike local autonomy and bottom-up social movements in new municipalism (Roth et al., 2023; Thompson et al., 2020), state entrepreneurialism demonstrates (party-)state centrality, especially central state centrality (Wu and Zhang, 2022). In residential neighbourhoods, party leadership is strengthened through ‘co-production’ (Li, 2022; Wang et al., 2024b). Unlike ‘challenging top-down narratives’ in municipal statecraft (Lauermann, 2018: 205), local governments are increasingly becoming the receiving end of systemic change to deliver extra-growth agendas. Although local governments in other contexts must absorb extra-territorial objectives (Angel, 2021; Beswick and Penny, 2018), the Chinese party-state bureaucratic system requires closer alignment with central mandates (Guo, 2020; Pearson et al., 2023; Wu et al., 2022). The central state has played a more prescribing role in transforming political mandates, guiding urban development agendas, and delivering changes on the ground.

Despite different positions, state entrepreneurialism and other forms of governance reflect the universal intertwining and tension in statecraft. Their contemporary transformation responds to evolving governance challenges in the global conjuncture of economic downturn, demand decline, territorial disputes, international backlash, and climate change. China’s particular territorial logic, originating from historical and geographical contingencies, is a crucial source of divergent statecraft. Post-pandemic economic downturn and geopolitical tensions have reinforced this territorial logic, particularly regarding national-states (Bremmer, 2010; Rolf and Schindler, 2023). The statecraft is increasingly directed by territorial political considerations, especially geopolitical tensions in various contexts in post-pandemic times (Lauermann, 2018; Peck, 2023; Wu and Zhang, 2022; Yeung, 2023).

## IV City statecraft of financialisation

In China’s urban development, local states attract market actors and devise their own market instruments to reap land values and stimulate the local economy (Wu, 2018, 2023). Recently, city statecraft has evolved to mobilise market instruments and financial tools to achieve extra-economic strategic goals as a response to strengthening territorial politics. This form of statecraft has accumulated a crisis of governance.

Urban development in China has been associated with land revenue maximisation and career advancement (Chien, 2013; He et al., 2018; He and Wu, 2009; Lin, 2014; Tao et al., 2010). The entrepreneurial nature of local governments in China is rooted in territorial logic, particularly the tax-sharing system and cadre promotion system. The tax-sharing system redefines the central-local relation in China by strengthening the tax revenue of the central state and restricting local tax revenue. Local governments are obligated to shoulder almost all the local development duties, including urban maintenance, infrastructure construction, and public welfare provision. To fill the fiscal voids, land-related income, such as land conveyance fees and property tax, is assigned to local income. Local governments are incentivised to explore land-based financial channels (Ong, 2014). Moreover, the cadre promotion system requires local cadres to conduct entrepreneurial activities for personal promotion (Guo, 2020). Hence, the tax-sharing and cadre promotion systems have urged local governments to explore financial conduits and act entrepreneurially (Lin, 2014).

As land is the most valuable resource manipulated by local governments, city statecraft centres on land (Hsing, 2010; Lin, 2014). Echoing research on the ‘entrepreneurial city’, entrepreneurialism has been used to illustrate land-centred urban development endeavours (Chien, 2013; He et al., 2018; Su, 2015). Local governments sell under-priced industrial land to attract investors while reaping long-term tax revenue and land appreciation (Tao et al., 2010), a critical way of driving local development (Lin, 2014; Su and Tao, 2017). Local governments not only attract external market investors but also devise their own market agencies, such as land development corporations (*chengtou*) (Jiang and Waley, 2018; Wu, 2018). Local governments transfer their land ownership or provide implicit guarantees to support land corporations to enter the financial market and explore various financial channels, such as bank loans, *chengtou* bonds and shadow banking products (Bai et al., 2016; Tsui, 2011). Hence, local borrowing is mainly backed by state-owned land (Pan et al., 2017).

Despite acting like entrepreneurs, local governments do not simply pursue revenue maximisation. Instead, entrepreneurial activities fuse together the state and the market to enable the state to act through the market (Wu, 2018, 2020). Urban entrepreneurial-like activities also carry strategic consideration beyond the city. For example, developing the Lingang New Town was connected to a strategic goal of building Shanghai as the ‘dragonhead’ of the Yangtze River region. To gain the support of four rural towns in this area, the municipal state allocated land to these towns rather than collectively managed it for appreciation. Lingang development also includes various development corporations. Their coalition was built up by economic considerations rather than command and control (Shen et al., 2020). The goal to achieve the strategic ambition tolerates capitalistic logic. Hence, local politics are not limited to growth coalitions but need to maintain the state power (Robinson et al., 2022; Wu, 2018).

Recently, local governments have faced increasing interference from upper-level governments to align with multiple agendas such as ‘Ecological Civilisation’, ‘People’s City’, and ‘Harmonious Society’ (Kostka and Zhang, 2018; Li and Zhong, 2021; Wu and Zhang, 2022; Zhang and Wu, 2024). Local entrepreneurial tactics are deployed to deliver extra-economic goals. On the one hand, local governments can utilise political priority to unlock local development resources, such as land quotas, specific subsidies, and offshore financing (Chung and Xu, 2021; Zhang et al., 2022a). On the other hand, local governments are responsible for delivering projects echoing new territorial politics. Local financial capacity based on land manoeuverers is challenged.

First, land-based financial techniques are stretched to secure funding for new territorial agendas. For example, in Chengdu, a greenway project was proposed to construct linear green spaces surrounding the metropolitan area, echoing the central initiative of ‘Ecological Civilisation’. The greenway project has been escalated as an urban development strategy to build a ‘park city’ (Zhang and Wu, 2024). The model combines the development of industrial and ecological spaces and was endorsed by President Xi Jinping in his visit in 2018. It is conducted by a local development corporation and financed by expected land appreciation (Zhang et al., 2022a). The Chengdu government established a greenway corporation to construct the greenway. The corporation expects to capture the spill-over effect on nearby land values to cover expenses in greenway construction. According to the plan of the Chengdu government, the corporation will receive one million yuan per *mu* from nearby land transactions from related district governments. These district governments also support the greenway project because they anticipate more land value appreciation after completion. Hence, the environmental objectives and incentives for land revenue are intertwined to deliver such a political project.

Second, new territorial agendas are not necessarily compatible with land-based entrepreneurial techniques, exacerbating tension between capitalistic and territorial logics. For instance, using land value capture to support environmental agendas may trigger local financial risks. Dali, a fourth-tier city in Western China, faces political pressure to protect the environment as President Xi Jinping commented on the protection of Erhai Lake in Dali. The protection project is mainly financed by a local government bond besides central subsidies. In 2019, Yunnan province (on behalf of the Dali government) issued a three-billion-yuan special local government bond, the first local bond against environmental projects. According to the regulations on issuing local government bonds, local governments should specify how they repay the bond. The Dali government demonstrated its solvency by arguing that land sales income from associated land plots would repay the bond. In this case, the land-backed financial tool is used not for financial (speculative growth) but for environmental ends because of territorial politics. However, the land sales income is far less than expected during the pandemic. The city government estimated to sell 245.61 *mu* land to get 1.86 billion yuan from 2020 to 2022 to repay the bond. In reality, it only received around one billion yuan from all the land transactions. Hence, whether the bond could be repaid by local land income is uncertain. The stagnancy of the real estate market, coupled with top-down political pressure on environmental protection, has jeopardised local fiscal health.

The issue of local financial risks is pervasive in China, rooted in the state’s responses to the crisis of capital accumulation. Since the stimulus package in 2008, local governments have been empowered to explore various financial conduits, such as *chengtou* bonds and trust bonds, through local development corporations. The shift towards financialised urban development reflected the deconstruction of the coherence of export-oriented industries in the aftermath of the global financial crisis. Still, the financial expansion has, in turn, led to new crises. These practices led to enormous local debts and increased land prices (Bai et al., 2016). Therefore, the central state has enacted regulations to control local finance and mitigate local financial risks. Local government bonds have been introduced to replace the previous land-backed implicit local borrowing channel to contain local debt, but the outcome is far from satisfactory (Li et al., 2023b; Wu, 2023). Local governments try to circumvent central control and maintain borrowing through development corporations. The rationale underneath does not mean that the central state cannot reshape local financing mechanisms. Instead, the central state is powerful in selecting and enacting local government bonds (Feng et al., 2024; Li et al., 2023b). The central state understands the necessity of mobilising local governments to pursue local development through domestic investment. This consideration gives leeway for local governments to manoeuvre. Development corporations can group with other local state-owned corporations to form a larger corporation group to exit from the central blacklist and maintain their financial function (Feng et al., 2023). Tension within territorial politics has pushed up local financial risks, warning of the sustainability of the ‘developing by borrowing’ model (Pan et al., 2017).

The alarming issue of local financial risks in post-pandemic China reflects the increasing tension. The central state tolerated the financial operations of local governments to support the economy and maintain social sustainability during the pandemic from 2020 to 2022. Therefore, the liabilities of local governments surged. In 2022, the outstanding balance of local government bonds was 35.06 trillion yuan, and the interest-bearing debt of local land corporations reached 50.2 trillion yuan. Meanwhile, the stagnant housing market has shrunk local land income, putting some local governments on the brink of insolvency. This is a crisis rooted in the end-of-20th-century housing reform, accentuated by the post-pandemic lack of demand and overlaid with a new crisis rooted in financialisation. To deal with the issue, the central state initiated a plan to comprehensively reduce the debt in 2023. The plan aims to swap hidden local debts (mainly from development corporations) with long-term refinancing bonds. By the end of 2023, 1.39 trillion yuan special refinancing bonds were issued. The financial burden of local governments is temporarily relieved by another round of state-led financial expansion. The financial risks persist rather than being resolved. The financial capacity of the local governments was reaffirmed as a pragmatic response to the pandemic, generating more financial risks, which finally exerted pressure on the state to deal with the new crisis.

To sum up, municipal governments have deployed innovative statecraft under strengthening territorial politics. China’s city statecraft is experiencing a similar ‘late-entrepreneurial’ moment (Peck, 2017), characterised by a trend of recentralising central control over local finance, multiple devolved tasks associated with hierarchical political considerations, and declining profitability of routine entrepreneurial endeavours. Local governments respond by deploying versatile market instruments and internalising finance into local operations. In doing so, city statecraft addresses economic and extra-economic strategic goals rather than being captured by the market (Wu, 2023). However, China’s city statecraft differs from ‘municipal statecraft’ in that state politics dominate the shaping and reconfiguration of city statecraft. The very form of statecraft accumulates the governance crisis, manifested in prolonged local financial risks.

## V Residential statecraft of the enterprising self

China’s shifting statecraft is also observed in residential communities. Working through not only market actors but also social organisations and active citizens, the state attempts to realise multiple strategic goals, including but not limited to development and stability.

The post-socialist state exercised governing technologies to govern at a distance (Zhang, 2010) and adopted entrepreneurial-cum-neoliberal tactics by motivating market actors – real estate developers and property management companies to manage residential estates and fulfil its developmental goals (Lu et al., 2019; Sun and Huang, 2016). Entrepreneurialism is also achieved through promoting autonomy, where the emphasis on self-responsible, self-organisation and community participation requires the making of the ‘enterprising self’ and the entrepreneurialisation of society (Hoffman, 2014). Social organisations, such as the homeowner’s association, have been introduced to organise community participation, adjudicate neighbourhood disputes and extend state’s infrastructure power (Cai and He, 2022; Fu and Lin, 2014; Wu, 2022), which reflects a greater capitalistic logic in the development and management of residential communities.

However, such a mode of entrepreneurial statecraft encountered a ‘latent crisis’ (Wan, 2016: 2344). Commercial development led to a rapid increase in disputes, contestations, protests, social conflicts, and a decline in social trust (e.g. Cao, 2022; Shin, 2013). Self-governance was not very successful (Cai and He, 2022). Residents were generally observed to be uninterested or indifferent to chances of participation (Heberer and Göbel, 2011). They refuse governments’ intentions to render them governable (Wan, 2016).

To address such crises, the Chinese state has diversified its governing strategies and promoted new state ethos and national political mandates that propel ‘new types’ of development, such as people-oriented development (Li and Zhong, 2021; Teo, 2023) and post-growth and high-quality development (Li, 2022; Wu et al., 2022). This is accompanied by an adjustment in local cadres’ career evaluation system that recognises the rising importance of non-economic factors and encourages a new round of ‘innovation tournament’ (Teets et al., 2017). Local governmental officials initiated governance experiments accordingly to pilot new approaches to deal with social pressures, cultivate governable subjects, and create new governance institutions, which ultimately aim to enhance the overall governance capacity of the state.

Apart from institutional adjustment, the statecraft adopted new participatory channels to encourage state-citizen interactions, such as co-action, co-design, and co-governance. This is especially true between the local state and a small group of active citizens and experts. For example, Teo (2023) highlights the role of ‘citizen intellectuals’ in Shenzhen’s urban village redevelopment experiment. Their participation connects the economic and extra-economic objectives of the state and contributes to ‘socially engaged municipal statecraft’. In a similar vein, Mai et al. (2023) reported the emergence of community gardens in Shanghai led by planning experts and non-governmental organisations. NGOs emerge as a new governing technique of the state to realise its extra-economic goals. This new technique reveals territorial logic in which NGOs play a significant role, bringing ‘an alternative subjectivity’ that moves ‘beyond both state authoritarianism and neoliberal urbanism’ (14).

However, both elements of the state (e.g. co-option) and entrepreneurial governance (e.g. expert-led and technocratic governance) are evident in the development of community gardens, suggesting that the new governing technique cannot be simplified as either capitalistic or territorial logic. The existing statecraft is a compromise out of dialectic tension between these logics – the authoritarian state deploying non-state actors. The recent statecraft with greater community participation in China is not necessarily a radical departure from entrepreneurialism. Instead, it embodies deeper engagement of societal actors and their entrepreneurial agencies under the state.

This new statecraft incorporates both territorial and capitalistic logic, as shown in the New Qinghe Experiment, which shows how the state and citizens interact in participatory regeneration on the ground (Wang et al., 2024b). The experiment was initiated in 2014 as a government-funded, expert-led social governance innovation programme to rejuvenate local communities and pilot new governance models through participatory regeneration.

First, entrepreneurialism is exercised with the help of experts and NGOs in the production of governable individuals and actionable communities during regeneration. They facilitated the formation of an ‘enterprising self’ through various forms of civic education and community participatory and volunteering activities, which (partially) empowered participants, built their capacities and enabled them to become the ‘true subject’ of space production (Jessop, 2022). Unlike traditional participation platforms, state agencies do not directly control these organisations and groups. Instead, they create new ‘citizen platforms’ where open participation is made possible, providing opportunities for ‘citizen intellectuals’ and a ‘public creative’ approach to the governance (Kochan, 2021; Teo, 2023). Rather than developing into ‘counter-power to the nation-state’ as advocated by radical municipalists (Roth et al., 2023), active community members emerged from these platforms and were often co-opted into the state-led governance networks. They were co-opted due to their abilities to self-mobilise, which ‘fit with the technologies of a broadly entrepreneurial script’ (McFarlane, 2012: 2798) outlined by the state.

However, directions of subject formation and boundaries of participation have been largely determined not by the community but by external actors, including experts and local governments. The involvement of experts thus reflects a broader shift towards professionalised and technocratic forms of participation. In this sense, participatory regeneration is post-political. It cultivates governable and acting subjects whose capacities and subject positions are in line with the intentions and objectives of the state. In doing so, potential tensions between the state and society are strategically reduced. In other words, the state realises its strategic goals through cultivating self-actualising subjects.

Second, the state (re)embeds itself into the community through social organisations. This is especially the case where initial attempts to cultivate acting and governable subjects fail (which is not rare), leading to the dominance of the territorial logic. For example, our longitudinal observation of the New Qing Experiment reveals the difficulty of maintaining the effects of participatory regeneration. Neither the new subjective positions (i.e. self-responsible and self-actualising communities) nor the new urban landscape (i.e. renovated community gardens) persist. Most community gardens that have gone through participatory regeneration deteriorated in a few years, generating criticism of the so-called do-it-yourself urbanism and its applicability in the Chinese context. To fix such a problem, the state mobilised its most ‘loyal’ followers – members of the CCP. They were tasked with tidying up the gardens and helping with maintenance issues regularly. Party-building is not just an ideological pronouncement or a political commitment but also an effective approach and instrument to achieve state objectives and strengthen territorial logic. Therefore, the participatory regeneration experiment does not lead to a process of community revitalisation. It starts with new approaches of entrepreneurial governance that work through and with (partially) empowered individuals and communities and evolves into an actually existing process of politicisation that reinforces the territorial logic of the party-state.

To sum up, we interpret China’s recent effort to govern urban neighbourhoods through new approaches to community participation as a new form of statecraft. This statecraft is characterised by a deeper process of social engagement, an extended form of entrepreneurialism, and stronger manifestations of territorial logic. It allows the state to act in more entrepreneur-like ways to govern urban communities by mobilising individuals, neighbourhood groups, and social organisations to become de facto entrepreneurs in everyday life who selectively employ entrepreneurial strategies to realise their own goals as well as the goals of the state. The emerging social and civic groups propagate participatory platforms and governance models that co-produce governable communities together with the local state in a socially engaged approach. When entrepreneurial approaches fail and ‘governable subjects’ are missing, CCP members, as both members of the society and agencies of the party-state, are mobilised in community participation, leading to a shifting process of re-politicisation that consolidates party-led territorial governance.

## VI Regional statecraft of spatial selectivity

Chinese statecraft has escaped from the ‘territorial trap’, and its geographical reach has exceeded beyond the traditional state-set territory of cities and increasingly engaged in cross-scale and cross-boundary politics (Brenner, 2019; Wu, 2016). For example, regional entrepreneurial governance has been gradually developed through inter-urban cooperation, which goes beyond the traditional territorial-bounded growth coalition centred on city-based development regime (Chien and Woodworth, 2018; Lin, 2014) and race-to-bottom inter-urban competitions. The ‘scaling up’ strategy through urban annexation has been identified as an essential instrument to mobilise land-based capital accumulation for the initial development and transformation of regional economies (Cartier, 2018; Li and Wu, 2018; Lin, 2009). More recently, the land development regime has been increasingly expanded through inter-city partnerships as an emerging driving force of city-region building (Zhang et al., 2023). The variegated rescaling process depicts the role of territorial politics in shaping city regionalism and forming a regional regime of capital accumulation.

City regionalism in China reflects the central state’s overarching bureaucracy, complex intra-state tension, and blurred state-market relationships. Despite local discretion and flexibility, local state agencies remain constrained within the institutional platforms devised by higher-level governments. Local state tends to align with the central state’s strategy due to the party-state’s political-economic system (Cartier, 2018; Lim, 2014). Since the 2000s, particularly under President Xi Jinping’s regime, reconfiguring the central-local relationship to ‘top-level design’ has resulted in changes in politics and governance at the macro level, showing a new trend of recentralisation. Regarding spatial strategies, the scale of city-region became a new form of state spatial selectivity, particularly with the inauguration of a new generation of regional strategies such as the collaborative development of the Beijing-Tianjin-Hebei (BTH) in 2014, Guangdong-Hong Kong- Macao Greater Bay Area (GBA), and the integrated development of the Yangtze River Delta (YRD) in 2019. In this sense, these state spatial strategies are ‘geographically variegated’ territorial reconfiguration driven by the party-state for extra-economic objectives that ultimately consolidate the CCP’s state power and legitimacy (Lim, 2014).

City regionalism has been orchestrated by the state for place-specific extra-economic objectives such as regionally coordinated development. Market mechanisms have been deployed and instrumentalised to achieve state-driven policy experimentations. Li and Wu (2018) detail the evolution of city-regionalism in China and highlight the different roles market mechanisms play for different state objectives. For example, initially extended urbanisation and economic regionalisation driven by market reform and global integration, regional planning practices for economic integration and coordinated development, internal state spatial selectivity and the geopolitical strategy of the nation-state. These driving forces can be subsumed into the interplay of territorial and capitalist logic to position the multi-layered and intertwined process of city regionalism, leaving little space for societal forces. Su and Lim (2023) similarly frame the relation between state interventions and capitalistic accumulation as a dynamic sovereignty-accumulation nexus. They argue that the political-economic evolution has been shaped by varying degrees of tension between capital mobility and territorial alliance since 1949.

The evolution is not a linear path towards an authoritarian state through recentralisation or a purely free-market state by neoliberalisation and decentralisation (Anguelov et al., 2023; Lim, 2014). Rather, it reflects the dialectical relationship as well as scalar differentiation at the global and sub-national scales (Lim, 2014). Regional statecraft in China is essentially state-orchestrated, determined by its specific state-market interplay under state entrepreneurialism (Wu, 2016). This evolution is contingent on the territorial logic of the party-state governance with varying associations with the capitalistic logic. The recurring territorial reconfiguration exemplifies a continuation of party-state-building ‘through negotiating the dialectical interactions between the territorial and capital logics of accumulation’ (Su and Lim, 2023: 710).

The regional plan and implementation of the BTH Coordinated Development is an example of the intra-state complexity, the contradictions between territorial logic and capitalistic logic, and the coexistence of different modalities of regional governance in post-reform China. Differences in regional economic foundation, political considerations and distinctive territorial logic make the BTH region different from China’s other mega-regions. The BTH region comprises two municipalities under the direct control of the central state (Beijing and Tianjin) and eleven cities in Hebei Province. The privileged political status of the capital city Beijing resulted in deeply uneven development and distributional inequalities, which functions as a ‘countervailing geopolitical’ force that leads to regulatory re-interventions at the city-regional scale (Li and Jonas, 2019). Beijing has transformed from a medium-sized socialist city in the 1980s to a mega-sized global city, with modern urban landscapes, dramatic physical expansion, a strong knowledge economy, as well as the emergence of informality and serious environmental issues (Mabin and Harrison, 2023). The rapid growth in Beijing at the expense of surrounding areas has also resulted in regional issues, such as regional disparities, spatial polarisation, environmental degradation, and hukou-based social exclusion. To cope with these challenges, President Xi Jinping proclaimed the coordinated development of the BTH region as a new national strategy in 2014, which reflects China’s latest strategic intention and constitutive practices of regional building.

City-regionalism is a result of regional statecraft of spatial selectivity (Wu, 2016). First, the new spatial strategic plan is scaled statecraft to cope with the socio-economic crisis resulting from deepened uneven development. The BTH region is primarily designed to solve Beijing’s ‘urban disease’, such as environmental issues and severe regional inequality. The coordinated development approach aims to reassert the role of Beijing as the national capital through the decentralisation of non-capital functions in relation to the political leader’s desire (Mabin and Harrison, 2023) and to promote the competitiveness and sustainability of BTH region as part of the nation-state’s geopolitical strategy (Li and Wu, 2018). The plan is rhetoric. It identifies three spheres of cooperation, namely, infrastructure, environmental, and industrial integration, but is ambiguous in terms of the accountability and operation details. This strategy indicates the ‘intransitive mode of governance’ that reproduces spatial/scale imaginary and materialises it through the party-state mobilisation (Meulbroek et al., 2023). Beyond mobilising the discursive spatial imaginaries, the central state also engages in emergent regional governance by making new policies and regulations and designating city regional projects and territorial distribution. For example, the central state designated and financially supported two high-profile spatial projects in implementing BTH coordinated development, namely, Tongzhou Administrative Sub-centre within Beijing and Xiong’an New Area in Baoding, Hebei, which are planned as the ‘two wings’ of Beijing. The spatial restructuring of Beijing is claimed as a spatial fix to satisfy the territorial and capitalistic logics simultaneously (Zou, 2022). Another example is the recent environmental city-regionalism observed in the BTH region. The central state drives city-regionalism through the state restructuring in the BTH for an eco-scalar fix to cope with the increasing environmental pressures and social concerns caused by PM_2.5_ crisis (Wang et al., 2023). Through the rescaling process, the central state can enhance its state control and governance capacity in terms of environmental and spatial regulations.

Second, zonal statecraft remains an instrument for coordinating economic and territorial development in the city-region (Anguelov et al., 2023). In the GBA, localised rezoning strategies that are layered on historical experimentations since the reform era co-function with (mega-)regional programmes as party-statecraft to pursue economic development and territorial management (Anguelov et al., 2023; Cartier, 2018). The (re)zoning strategies are more ambitious in the BTH region, including creating a new city (the Xiong’an New Area) and a new administrative sub-centre of Beijing (Tongzhou). Non-capital functions such as research institutes and universities, municipal government departments, state-owned enterprises (SOEs), and hospitals have been mobilised by the central state and Beijing Municipal Government to relocate to Tongzhou and Xiong’an in a top-down fashion. To achieve its strategic goals, the central state creates new state apparatus and market-like entities beyond the existing administration system. These new zones are operated in the sophisticated development model, the ad hoc management committee model, with affiliated state-owned development corporations.

Here, the state’s territorial logic prevails over capitalistic logic. Regional projects are designated for extra-economic objectives such as coordinated development in cross-boundary areas, policy experimentation, and solving social and ecological issues rather than merely creating new space for capital accumulation. For instance, the development of the Tongzhou Administrative Sub-centre is not confined within its district jurisdictional area. It aims to bolster the development of three neighbouring counties in Hebei, namely, Sanhe, Dachang, and Xianghe. This cross-boundary area is designated as a demonstration zone for the coordinated and high-quality development of the BTH region. Within the demonstration zone, the jurisdictional boundary is defined to create a cohesive territorial unit by promoting unified planning, policies, standards, and administration.

Third, vertical and horizontal collaborations within the state facilitate the materialisation of city regions to realise territorial agendas. The state power is not homogenous but varies across different scales of government. The grounded process of city-region building in China is not orchestrated by the nation-state from above, a growth coalition formed from below, or a simple division of labour between the central and local governments (Li et al., 2023a; Zhang et al., 2023). Instead, two intertwined processes, including centrally orchestrated regional imaginary and regional cooperation through multi-scalar alliances, co-produce city regions under state entrepreneurialism. The region building involves a scalar division of administration, and the actual implementation depends on local governments (Su and Lim, 2023). To align with the national political mandates or strategies (Wu et al., 2022), local governments increasingly enforce top-down initiatives or intertwine their own development agenda with national strategies through new spatial practices and institutional innovation, owing to the path dependency on the hierarchical political system and the strengthened national-state intervention or party-state discipline during the rescaling process.

To sum up, city-regionalism, spatial imaginary and the constitutive practices, including versatile modes of zoning and trans-jurisdictional projects, are together embedded in the modalities and spatiality of the party-statecraft for political, economic, social, and environmental ends (Anguelov et al., 2023; Li et al., 2023a; Meulbroek et al., 2023). In this sense, the regional statecraft is developed in conjunctural and relational processes of intertwined top-down and bottom-up approaches (Zhang et al., 2022b). China’s latest city-region building attempts reflect the transformation of regional statecraft from inter-city competition and fragmented administrative division to an ongoing process of cooperation and regionalisation (Li et al., 2023a). The spatial imaginary envisioned by the central state mobilises and orchestrates the scaled actions and interests, and concrete projects are designated either by the central state directly or through multi-scalar alliance and coalition building. This form of regional statecraft reflects the state’s strategic intentions to ease regional inequality and cope with the emergent social and environmental crises and changing geopolitics through various state-led spatial and scalar fixes. In this process, the territorial logic prevails, and the capitalistic logic is strategically incorporated to consolidate the territorial logic and strengthen party-state power. However, implementing the agenda faces challenges due to the contradiction between territorial and capitalistic logics (Li and Wu, 2018; Su and Lim, 2023). The inherently coherent agenda is turned into selective upscaling and ad hoc customised governance arrangements.

## VII Conclusion

The grounded view from China reveals the pervasive capitalistic logic with versatile entrepreneurial activities and long-lasting public-sector endeavour for economic growth. But the Chinese story is also about statecraft – in response to geopolitical challenges and opportunities, there is a changing art of conducting state affairs. Such an intertwined, combinational and contradictory nature has been widely noted in the literature (Harvey, 2005; McNally, 2020; Peck, 2021, 2023; Wu, 2018), often denominating as ‘Chinese characteristics’. The recent literature on state capitalism highlights a universal presentation, rather than particularity, of late capitalism (Alami and Dixon, 2023; Sperber, 2022). In this sense, China is less exceptional – what is special is a particular way of introducing marketisation through a historical process. Its market operation is constrained but reflects statecraft for the first instance – entrepreneurialism as statecraft in both state ownership and emerging private sectors. It is a historical continuation of ‘market in state’ (Zheng and Huang, 2018) and a ‘strategic intent’ which guides the economy through the art of conducting state affairs (Weber, 2023; Wu, 2018).

This paper reveals Chinese statecraft in examples of city development and residential and regional governance, as shown in their varieties worldwide (Lauermann, 2018; Phelps and Miao, 2020; Pike et al., 2019; Thompson, 2021). Rather than showing whether Chinese statecraft aligns with or contrasts with any singular form discovered elsewhere, we position this Chinese view and other statecrafts (plural form) together in a spectrum of different combinations of capitalistic and territorial logics. We demonstrate how these resonate with global transformative governance. This enables clarification and positioning of Chinese statecraft as incorporating both logics, showing a particular combination of historical and geographical contingencies, and demonstrating universal tension between the two logics in the conjunctural moments of late capitalism and geopolitics.

Chinese urban development strategies represent the art of mobilising capital through the land (Feng et al., 2022; Wu, 2023). The actual management involves the assemblage of trans-local state actors (Shen et al., 2020; Su and Lim, 2023; Wang et al., 2024a) but is also subject to a multi-scalar state mandate like Ecological Civilisation and the ‘People’s City’ (Kostka and Zhang, 2018). The city statecraft deals with both economic and extra-economic objectives (Lauermann, 2018) and development corporations serve a purpose more than capturing land rent but also state strategic agendas such as ‘indigenous innovation’ (Zhang and Lan, 2023; Zhu et al., 2024), especially in today’s transforming global production networks and new international geopolitics. In its own ways, China experiences a late entrepreneurial moment with stronger state intervention and social movement (Peck, 2017; Thompson, 2021; Thompson et al., 2020).

Residential statecraft tends to mobilise and co-opt the community and society beyond fostering neoliberal subjectivity (Mai et al., 2023; Zhang and Ong, 2008). The recent emphasis on ‘co-production’ and ‘co-governance’ demonstrates more than continuing authoritarianism into everyday life. Rather, it is the social management of state entrepreneurialism. Instead of managing the community by the state, the statecraft transforms itself into ‘governing *with* communities’ in the name of ‘co-governance’. What is salient in the Chinese script, different from radical municipalist (Roth et al., 2023), is the role of the Chinese Communist Party (Cartier, 2018; Li, 2022; Pearson et al., 2023). Therefore, the ‘entrepreneurial’ script stresses public management innovation through market self-regulation, making the enterprising self and party-guided social mobilisation.

In a larger sub-national territory, a territorial strategy of the party-state stresses inter-jurisdictional coordination and a unified market for a geopolitical agenda (Li and Jonas, 2019). As a statecraft managing its strategic space, state rescaling presents a ‘geopolitics of city regionalism’ (Jonas and Moisio, 2018) beyond local growth coalition and growth machine (Brenner, 2019). Besides centrally orchestrated regional imaginary and regional cooperation through multi-scalar alliances (Li et al., 2023a), China’s city-region building incorporates both territorial and capitalistic logics (Anguelov et al., 2023; Su and Lim, 2023; Zou, 2022), a ‘zoning strategy’ of party-state (Anguelov et al., 2023), institutional collaboration (Wang et al., 2023), and state experimentalism. The large-scale ‘zoning’ experiments, such as Beijing’s capital region and the Greater Bay, show a different interpretation from ‘zoning as an exception’ of state sovereignty (Ong, 2007). It is statecraft managing the economic affairs of its territory into ‘territorial economies’ (Cartier, 2018) and the management of space on a grand scale in an extended and even planetary form through state-scale selectivity (Brenner, 2019; Wu, 2016).

What is original in this China story is not its unique statecraft as exceptional state authoritarianism but rather its nature of frontier of capitalism where, despite such a planetary nature of capitalist urbanisation, capitalistic logic confronts territorial logic, shaping universally existent but versatile statecraft. By this, we abstract the grounded experience of Chinese statecraft as ‘state entrepreneurialism’ that represents a particular combination of capitalistic and territorial logics. That is, Chinese statecraft maintains state strategic and extra-economic intention through deploying and mobilising market and society – to create its own agents and to co-opt those that are already existent or emerging. Recent changes in late capitalism and geopolitics have reinforced the dependency on dominant territorial politics, particularly in China, modernisation as a national-state strategy, the CCP’s centrality in politics, and local leadership’s upward accountability politics. Political mandates increasingly steer urban development and regional governance. Residential statecraft mobilises society to consolidate party leadership through innovative and experimental approaches.

State entrepreneurialism conceptualises such territorial politics centrality not as transcending economic structure but as conditioned by global capitalist dynamics. As China is increasingly crucial in global production networks, statecraft takes the benefits and risks. Innovative neighbourhood experiments, local state agencies, and central state mandates are constrained by financing balance sheets and accumulation dynamics, leading to severe local debts, similar to austerity urbanism (Peck, 2012). This conceptualisation makes possible an alternative to a particular form of pro-growth neoliberal entrepreneurialism, as China is facing another turn in post-pandemic governance. Chinese statecraft delinks a pro-accumulation and pro-growth agenda, not from local autonomy and everyday life (Lauermann, 2018; McFarlane and Silver, 2017; Thompson, 2021; Vincent, 2023) but from party leadership and state mobilisation (Wu, 2022; Wu and Zhang, 2022). From reinterpreting city statecraft, residential statecraft, and regional statecraft, we find that the turn to neoliberalism has not been full-fledged, even though the market forms have been aggressively imposed. Its party-state-dominated statecraft might present a contrast to financialisation dominated by elite investors and governance of elite capture (Lauermann and Mallak 2023). Still, both raise attention to the statecraft at the core of future imagination and narratives of urban governance.
